# Regulation of p53 by the mitotic surveillance/stopwatch pathway: implications in neurodevelopment and cancer

**DOI:** 10.3389/fcell.2024.1451274

**Published:** 2024-09-27

**Authors:** Travis H. Stracker

**Affiliations:** Center for Cancer Research, Radiation Oncology Branch, National Cancer Institute, Bethesda, MD, United States

**Keywords:** p53, USP28, 53BP1, cancer, c-MYC, neurodevelopment, microcephaly, apoptosis

## Abstract

The transcription factor p53 (encoded by *TP53*) plays diverse roles in human development and disease. While best known for its role in tumor suppression, p53 signaling also influences mammalian development by triggering cell fate decisions in response to a wide variety of stresses. After over 4 decades of study, a new pathway that triggers p53 activation in response to mitotic delays was recently identified. Termed the mitotic surveillance or mitotic stopwatch pathway, the USP28 and 53BP1 proteins activate p53 in response to delayed mitotic progression to control cell fate and promote genomic stability. In this Minireview, I discuss its identification, potential roles in neurodevelopmental disorders and cancer, as well as explore outstanding questions about its function, regulation and potential use as a biomarker for anti-mitotic therapies.

## Introduction

The transcription factor p53 (encoded by *TP53*) is one of the most well studied tumor suppressors and the most frequently mutated gene across human cancers (Oren and Prives, 2024). Mutations in p53 can lead to both loss or gain of function effects and hereditary p53 mutations underlie the cancer predisposition syndrome Li Fraumeni (McBride et al., 2014). P53 plays a critical role in the activation of cell fate decisions in response to many different stresses, including DNA damage, nutrient deprivation, oxidative and ribosomal stress (Peuget et al., 2024). P53 undergoes extensive transcriptional, post-transcriptional and post-translational regulation that control its levels and functions in different cell types (Hafner et al., 2019). In the absence of stress, p53 levels are low, in large part due to the activity of MDM2, a ubiquitin ligase that targets p53 for proteosomal degradation and prevents its transcriptional activity (Peuget et al., 2024; Hafner et al., 2019).

Often referred to as the “guardian of the genome,” p53 is an integral part of the DNA damage response (DDR) (Hafner et al., 2019; Abuetabh et al., 2022). The loss of p53 in cancer allows for the tolerance of DNA damage and is associated with high levels of chromosomal instability. In response to DNA damage, p53 is activated and stabilized to control cell cycle progression, regulate DNA repair and control cell fate decisions, including apoptosis and senescence, that are both important tumor suppressive mechanisms (Hafner et al., 2019; Abuetabh et al., 2022). The full activation of p53 in response to DNA damage requires the activity of the ATM and CHK2 kinases, both of which are mutated in cancer predisposition syndromes, as well as many sporadic cancers (Hafner et al., 2019; Abuetabh et al., 2022; Smith et al., 2020). Targets of ATM and CHK2 include p53 and its negative regulators, MDM2 and MDM4. The DDR induced activity of p53 is also negatively regulated by WIP1 (encoded by *PPM1D*), a p53-activated protein-phosphatase that targets the activating phosphorylation events deposited by ATM and CHK2 (Zhu and Bulavin, 2012).

**FIGURE 1:**
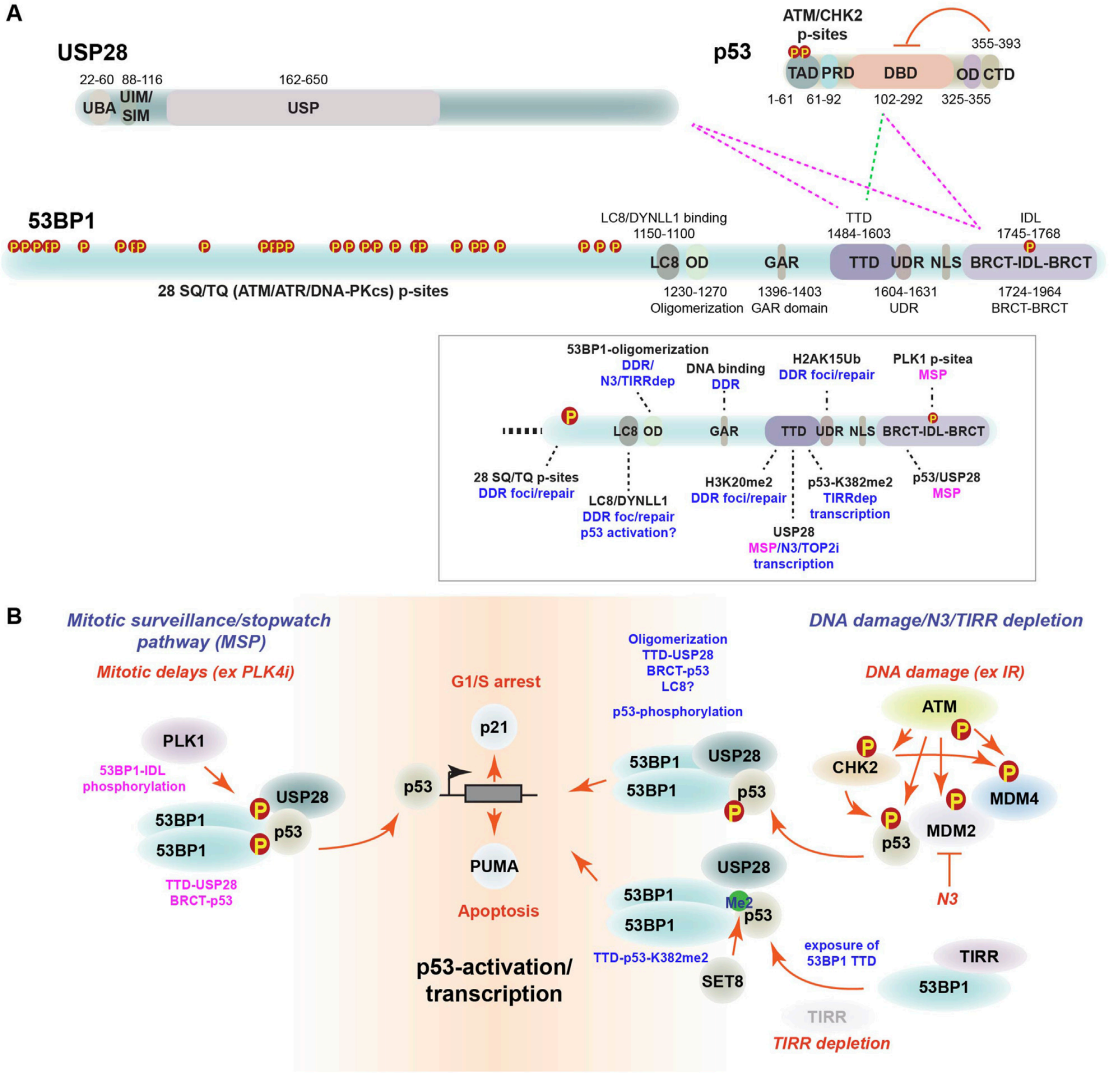
The MSP complex. **(A)** Schematic p53, USP28 and 53BP1 protein domains. ATM and CHK2 kinase phosphorylation sites, transcriptional activation domain (TAD), proline rich domain (PRD), oligomerization domain (OD) and C-terminal domain (CTD) are shown for p53 with amino acids indicated. The ability of the CTD to negatively regulate the DBD is indicated (red lines). The Ubiquitin Associated domain (UBA), ubiquitin interacting motif (UIM), SUMO interacting motif (SIM) and ubiquitin specific protease (USP) domains are shown for USP28. The LC8/DYNLL1 binding domain (LC8), oligomerization domain (OD), glycine-arginine rich (GAR) domain, tandem Tudor domain (TTD), ubiquitin-dependent recruitment motif (UDR), nuclear localization signal (NLS), BRCA C-terminal domains (BRCT) and intrinsically disordered loop (IDL) are indicated for 53BP1. Dotted lines indicate 53BP1 domains implicated in interactions with USP28 and p53 in the MSP complex (pink) or following TIRR depletion (green). The box illustrates the pathways (blue) in which specific domains have been implicated with the domains involved in the MSP highlighted in pink (Meitinger et al., 2024; Fong et al., 2016; Rass et al., 2022; Cuella-Martin et al., 2016; Cuella-Martin et al., 2021; Parnandi et al., 2021; Lo et al., 2005; Picart-Armada et al., 2017). TIRR depletion is indicated as TIRRdep. **(B)** Roles of the MSP complex. The MSP (left) is triggered during prolonged mitosis by PLK1 phosphorylation of the 53BP1 IDL (Meitinger et al., 2024). Subsequent formation of inherited MSP complexes with USP28 binding to the 53BP1-TTD and p53 binding to the 53BP1-BRCT domain leads to activation of p53 target genes in G1 to control cell fate decisions. DNA damage-mediated activation of p53 (right) involves its phosphorylation by ATM and CHK2, as well as inhibition of negative regulators MDM2/MDM4 and WIP1 (not shown). The LC8 interaction domain has been implicated in p53 nuclear localization after damage but its role in transcription remains undefined (Lo et al., 2005). Stabilized, phosphorylated p53 interacts with the 53BP1 BRCT domain. N3 inhibition of MDM2 activates p53 via a similar mechanism but appears to bypass the need for DDR kinase activity. Finally, depletion of TIRR activates p53 in a manner requiring interactions between the 53BP1-TTD and p53-dimethylated on K382 (bottom right) that require USP28 in a yet to be defined role (Parnandi et al., 2021).

Mutations in p53 cluster in hotspots that include its DNA binding domain (DBD) and inherited p53 mutations underlie the Li Fraumeni cancer predisposition syndrome (Figure 1A) (McBride et al., 2014). Li Fraumeni patients are predisposed to many cancer types, including tumors of the bone, breast, brain and skin. Despite its central role in tumor suppression, many cancers have intact p53. Frequent loss of p53 activating kinases, including ATM and to a lesser extent CHK2, are observed in many cancer types (Smith et al., 2020). Similarly, amplification of p53’s negative regulators MDM2 and MDM4 were implicated in tumorigenesis through their effects on p53 levels (Sun et al., 2024; Wu et al., 2021).

In addition to cancer, p53 plays an important role in mammalian development with crucial roles in the central nervous system. Deletion of negative regulators *Mdm2* or *Mdm4* in mice led to p53 activation and early embryonic lethality that could be rescued by p53 deletion (Wang and Attardi, 2022). Numerous mutations in p53 that lead to its hyperactivation have been linked to a wide spectrum of developmental phenotypes in mice, including exencephaly, cerebellar hypoplasia, craniofacial defects, hematopoietic defects and microcephaly or reduced brain size [see reference (Bowen and Attardi, 2019) for a detailed overview]. Recent reports identified *de novo* mutations in p53 that truncate its C-terminal domain (CTD), that negatively regulates its DBD, in patients with microcephaly, growth defects and bone marrow failure (Figure 1A) (Toki et al., 2018). Abundant evidence from mouse models also established a key role for aberrant p53 activation in phenotypes resulting from mutation or loss of genes involved in several key cellular processes, including the DNA damage response (ex. *Brca1, Xrcc4, Lig4*, *Nbn*, *Xrcc2*, *Fancd2, Smc5*), telomere maintenance (ex. *Acd, Terc*), centrosome duplication (ex. *Aspm*, *Cenpj*, *Cep63, Sas4*) and translational regulation (ex. *Tcof1, Rps7, Rps19*) (Bowen and Attardi, 2019; Atkins et al., 2020). In addition, aberrant p53 activation was implicated in the pathology of several human syndromes including Nijmegen Breakage Syndrome, Diamond-Blackfan Anemia, Fanconi Anemia and Dyskeratosis Congenita (Wang and Attardi, 2022; Bowen and Attardi, 2019).

## Identification of the mitotic surveillance/stopwatch pathway (MSP)

Centrioles are microtubule-based structures that recruit the pericentriolar material composed of over a hundred proteins to form the centrosome (Nigg and Holland, 2018). Centrosomes are the primary microtubule organizing centers and their duplication occurs in a cell cycle dependent manner in parallel to genome duplication. This process is tightly controlled to ensure a single duplication event takes place resulting in 2 centrosomes to form the mitotic spindle (Nigg and Holland, 2018). Defects that lead to supernumerary centrosomes or centrosome loss, can slow mitotic progression and impair mitotic fidelity (Lens and Medema, 2019; Levine and Holland, 2018). The spindle assembly checkpoint (SAC) delays anaphase onset until the kinetochores of all the chromosomes have attached to the mitotic spindle and is one of the primary mechanisms by which mitotic time is extended (see review for more details) (McAinsh and Kops, 2023). Seminal studies in non-cancer cells used anti-mitotic agents, such as taxols, to prolong mitosis, revealing that over a certain time threshold, they activated p53 and initiated a p53-dependent G1 arrest in daughter cells in the absence of chromosome segregation errors or detectable DNA damage (Uetake and Sluder, 2010). However, it remained mechanistically unclear how p53 was activated in this context.

The protein kinase PLK4 plays a conserved role in centriole duplication and genetic perturbation or inhibition of PLK4 with centrinone, a small molecule inhibitor, elicited centriole loss, mitotic delays and a p53-dependent G1/S cell cycle arrest, reminiscent of the effects of taxols (Nigg and Holland, 2018; Lambrus et al., 2015; Wong et al., 2015). While non-transformed cells normally completed mitosis within ∼30′, extending mitosis to ∼90–120′ with centrinone or other agents led to an irreversible G1/S arrest in daughter cells (Lambrus et al., 2015; Wong et al., 2015; Meitinger et al., 2024). Notably, this also occurred in the absence of DNA damage markers like γH2AX, a marker of DNA double-strand breaks (DSBs), or phosphorylation of p53 that is carried out by the DNA damage activated kinases ATM and CHK2, respectively (Hafner et al., 2019; Abuetabh et al., 2022). Many cancer cells treated with centrinone showed only a moderate reduction in cell growth, likely reflecting the p53-dependence of this cell cycle arrest pathway (Wong et al., 2015; Meitinger et al., 2024).

Multiple groups examined the genetic determinants of PLK4 inhibitor sensitivity using CRISPR/Cas9 based screening approaches, identifying a short list of proteins that included p53, its transcriptional target p21, a CDK inhibitor with a well-defined role in the G1/S transition, as well as two proteins previously implicated in the DDR, USP28 and 53BP1 (Figure 1A) (Fong et al., 2016; Lambrus et al., 2016; Meitinger et al., 2016). This new USP28-53BP1-dependent pathway that activated p53 in response to mitotic delays was termed the *mitotic surveillance or mitotic stopwatch pathway* (MSP) (Fong et al., 2016; Lambrus et al., 2016; Meitinger et al., 2016).

The identification of USP28 and 53BP1 as the central players of the MSP was initially puzzling, as both were implicated in the DDR that was inactive in response to centrinone (Lambrus et al., 2015; Wong et al., 2015; Fong et al., 2016; Lambrus et al., 2016; Meitinger et al., 2016). Further interrogation of 53BP1 in the MSP revealed that its tandem Tudor domain (TTD) mediated interactions with USP28, while its tandem BRCT domain was required for p53 interactions, demonstrating differences between the roles of these domains in DNA repair, where the TTD engages H3K4me20 and the BRCT domain is largely dispensable (Figure 1A) (Meitinger et al., 2024; Rass et al., 2022). Notably, PLK4 overexpression leads to centrosome amplification, that also triggers p53 activation (Marthiens et al., 2013). However, this was not rescued by the loss of USP28, indicating that it is a molecularly distinct p53-activation pathway (Evans et al., 2021).

Many drugs that activated the MSP, including taxols, Monastrol (Eg5 kinesin inhibitor) and PLK4 inhibitors, also chronically activate the SAC, suggesting potential signal integration between the MSP and SAC (Lambrus et al., 2015; Wong et al., 2015; Fong et al., 2016; Lambrus et al., 2016; Meitinger et al., 2016; Mayer et al., 1999). However, no components of the SAC were identified in screens for MSP components, and elegant experiments using the inducible depletion of the Comet protein, that results in slower anaphase without affecting chromosome attachment or spindle assembly, also activated the MSP (Meitinger et al., 2024; Fong et al., 2016; Lambrus et al., 2016; Meitinger et al., 2016). Together these data indicate that the MSP can function independently of the SAC, but extended mitotic time resulting from SAC activation is likely a major driver of MSP activation in some contexts.

53BP1 was originally identified as a p53 binding protein and was shown to promote p53 transcriptional activity (Iwabuchi et al., 1998; Iwabuchi et al., 1994). Structural studies later established the details of the interaction between p53 and the BRCT domains of 53BP1 (Figure 1A), although the functional significance of the interaction remained unclear until recently (Derbyshire et al., 2002; Joo et al., 2002). Since its original identification, a large body of literature established 53BP1 as a key regulator of DNA repair pathway choice, a role that is independent of its p53 interaction (Cejka and Symington, 2021). 53BP1 antagonizes BRCA1 to restrain nucleolytic end-resection and promote the use of the non-homologous end-joining pathway (Cejka and Symington, 2021). 53BP1 recruitment to damage involves the recognition of the dual histone marks H4K20me2 by the 53BP1 (TTD) and H2AK15ub by the ubiquitin dependent recruitment (UDR) domain, as well as the recruitment of additional proteins including RIF1 and the Shieldin complex consisting of SHLD1/2/3 (Figure 1A) (Cejka and Symington, 2021). During immune system development, 53BP1 is required for efficient class switch recombination, but dispensable for V(D)J recombination due to redundancy with XLF/NHEJ1 (Kabrani et al., 2023). Genetic crosses of 53BP1 deficient mice with p53 null mice revealed additional phenotypes, including enhanced ionizing radiation (IR) sensitivity, increased lymphomagenesis and elevated chromosomal instability, indicating that 53BP1 has many important functions that are independent of p53 (Morales et al., 2006; Ward et al., 2005).

USP28 is a ubiquitin serine protease that was first identified by homology searches with other ubiquitin serine proteases and later implicated in the DDR through protein interaction studies that identified a number of DDR related proteins including 53BP1, NBS1 and CHK2 (Zhang et al., 2006; Valero et al., 2001). Subsequent studies found that USP28 was recruited to DNA damage in a manner that required the tandem BRCT domains of 53BP1 (Knobel et al., 2014). However, USP28 loss did not strongly impair DDR signaling or DNA repair, consistent with previous work that found the 53BP1-BRCT domains were not required for the DNA repair functions of 53BP1(Figure 1A) (Knobel et al., 2014; Ward et al., 2006). Genetic deletion of USP28 in mice did not identify any strong DDR defects and did not impair IR induced apoptosis that requires ATM, CHK2 and p53 (Knobel et al., 2014). In addition to its role in the DDR, USP28 was also demonstrated to stabilize the c-MYC oncogene by antagonizing its ubiquitin-mediated degradation by the ubiquitin ligase FBW7 that is frequently lost in cancers (Popov et al., 2007a; Popov et al., 2007b).

## Variations on a theme in p53-transcriptional activation

Contemporary studies also identified a role for 53BP1 and USP28 in p53-mediated transcription in response to Nutlin-3 (N3), a small molecule antagonist of MDM2, and DNA damage (Brummelkamp et al., 2006; Cuella-Martin et al., 2016). 53BP1 KO cells showed reduced sensitivity to N3 and had a blunted p53-dependent transcriptional response following N3 or IR treatment (Cuella-Martin et al., 2016). Complementation mapping of the key domains involved in N3 sensitivity identified the oligomerization domain (OD) and BRCT domains of 53BP1, that had previously been shown to recruit USP28 to sites of DNA damage (Figure 1A) (Knobel et al., 2014; Cuella-Martin et al., 2016). The use of a 53BP1 BRCT mutant demonstrated that the DNA repair and USP28 recruitment functions were independent, as BRCT mutants conferred radioresistance, similar to p53 loss, in contrast to 53BP1 KOs that were radiosensitive due to DNA repair defects (Cuella-Martin et al., 2016). Structural and functional analysis further implicated the BRCT domains of 53BP1 in mediating interactions with both p53 and USP28 to transcriptionally activate p53-target genes and the G1/S checkpoint independently of DNA repair. Notably, a mutation in the 53BP1 TTD gave an intermediate phenotype, suggesting that the recognition of a methylated protein may contribute to the response (Cuella-Martin et al., 2016). A CRISPR base editing analysis of 53BP1 found that the TTD domain interacted with USP28 and was important for transcriptional responses to N3 or Doxorubicin, a Topoisomerase 2 (TOP2) inhibitor that induces DSBs, but the TTD was not required for DNA repair (Cuella-Martin et al., 2021). 53BP1 is also negatively regulated by the TIRR protein that masks the TTD, preventing its recognition of H4K20me2 and potentially other interactors (Drane et al., 2017; Zhang et al., 2017). Depletion of TIRR hyperactivated the p53 transcriptional response by allowing interactions between the 53BP1 TTD and p53-K382me2 in the p53 CTD (Figure 1A) (Parnandi et al., 2021). Transcriptional activation of p53 by TIRR depletion also required USP28, although the domain interaction requirements for USP28 in this context were not elucidated.

Collectively, these studies all identified 53BP1 and USP28 as key proteins for efficient p53-dependent transcription in response to mitotic delays, MDM2 inhibition (N3), TIRR depletion and DNA damage (TOP2 inhibitors and IR) (Figure 1B). However, they appear to rely to some extent on distinct protein domains, interactions and signaling inputs, the precise details of which remain to be further defined. The MSP, as well as the transcriptional response to N3 or DSBs (IR or TOP2 inhibition) requires interactions between the 53BP1-TTD and USP28, as well as the 53BP1-BRCT and p53 (Figure 1A). Both N3 and DNA damage-mediated transcriptional activation of p53 also rely on the 53BP1-OD that has not been tested in the response to mitotic delays to my knowledge (Figure 1A). The p53-transcriptional response to DSBs requires ATM-CHK2-mediated phosphorylation of p53 that is dispensable for the MSP, N3 and TIRR depletion responses (Cuella-Martin et al., 2016; Parnandi et al., 2021; Stracker et al., 2008; Phan et al., 2021; Marjanovic et al., 2015). Activation of p53-transcription by TIRR depletion relied on 53BP1-TTD interactions with p53-K382me2 and USP28, however it remains unclear if USP28 interacts with 53BP1 in this context (Parnandi et al., 2021). One possibility is that the interactions identified under different conditions all represent different stages of the same pathway. For example, the activation of the MSP may require initial interactions between the 53BP1-TTD and USP28 that transition into USP28 displacement and TTD engagement of p53-K382me2 that was recruited by the 53BP1-BRCT domain prior to chromatin binding and transcriptional activation in G1. Further experiments will be needed to establish the full extent of the similarities and differences in the mechanisms by which these distinct inputs activate the 53BP1-USP28-p53 axis to control p53-dependent transcriptional responses.

## Regulation of the mitotic surveillance pathway

As the activation of the DDR was not required to initiate the MSP, understanding how the pathway is initiated and regulated remains important to determine. Previous work showed that 53BP1 localized to kinetochores in mitotic cells and was hyperphosphorylated after treatment with nocodazole, a microtubule depolymerizer that prevents mitotic progression (Jullien et al., 2002). The kinetochore is a major site for the localization of SAC components that can extend mitotic time by delaying anaphase onset when kinetochores lack microtubule attachments to the mitotic spindle (McAinsh and Kops, 2023). Mitotic phosphorylation of the 53BP1 UDR domain was shown to inhibit DNA repair to prevent telomere fusions, indicating that the mitotic functions of 53BP1 were likely independent of its role in DNA repair (Orthwein et al., 2014). Together, this suggested that the kinetochore pool of 53BP1 could potentially be monitoring mitotic timing via interactions with these sites of attachment of the chromosomes to the mitotic spindle. Affinity purification of 53BP1 from mitotic cells revealed the kinetochore protein CENP-F as a prominent interactor and subsequent experiments demonstrated its requirement for 53BP1 localization to kinetochores (Burigotto et al., 2023). However, loss of CENP-F and the resulting perturbation of 53BP1’s kinetochore localization did not impair MSP activation of p53 (Orthwein et al., 2014). Subsequent investigation of kinase dependent kinetochore localization identified a role for PLK1 in the removal of 53BP1 from kinetochores, as PLK1 inhibitors, or the use of analog sensitive PLK1 mutants, resulted in 53BP1 retention. Using a proximity-ligation assay approach in mitotically arrested cells, it was observed that PLK1 inhibition prevented the association of 53BP1 with p53, strongly impairing the activation of the MSP. However, the mutation of 13 candidate PLK1 sites in 53BP1 did not impact its kinetochore localization, suggesting that 53BP1 was potentially not the direct target of PLK1 in the MSP.

A subsequent study also identified PLK1 as a key regulator of the MSP, but in contrast to the previous work, concluded that 53BP1 was indeed a direct PLK1 target in the MSP (Meitinger et al., 2024). Mutating 35 candidate PLK1 sites, they identified 3 sites in the IDL domain of 53BP1 between the tandem BRCT domains (Figure 1A) to be required for p53 interactions and MSP function. Using a live cell imaging approach, they observed that non-transformed cells treated with Monastrol exhibited mitotic delays of varying durations. Single cell tracking revealed that the cells that underwent a prolonged mitosis above a certain threshold, exhibited elevated p21 levels and G1 arrest in the daughter cells. MSP activation was proportional to the extent of the delay, as cells with longer mitotic delays inherited more MSP complexes. Moreover, it was integrated over time, as multiple interventions that prolonged mitotic progression below the arrest threshold, over multiple cell cycles, led to the accumulation of higher p21 levels and eventual cell cycle arrest. Analysis of cell cycle dependent MSP complex formation showed that the 53BP1-USP28-p53 interaction was specific to mitosis. Even after DNA damage, where USP28 recruitment to 53BP1 foci is visible, a stable MSP complex was not detected and mutations in the BRCT or TTD domains of 53BP1 selectively disrupted the p53 and USP28 interactions, respectively (Meitinger et al., 2024; Knobel et al., 2014). This striking “analog memory” model posits that during extended mitoses, soluble MSP complexes accumulate in a manner promoted by PLK1-mediated phosphorylation of 53BP1 and provide daughter cells with an analog memory of the events in the form of accumulated 53BP1-UPS28-p53 complexes. Entry into G1 allows the chromatin binding of p53 and activation of a G1/S arrest via p21 induction in daughter cells that is proportional to the extent of the previous mitotic delays.

## The role of the MSP pathway in neurodevelopment

**FIGURE 2:**
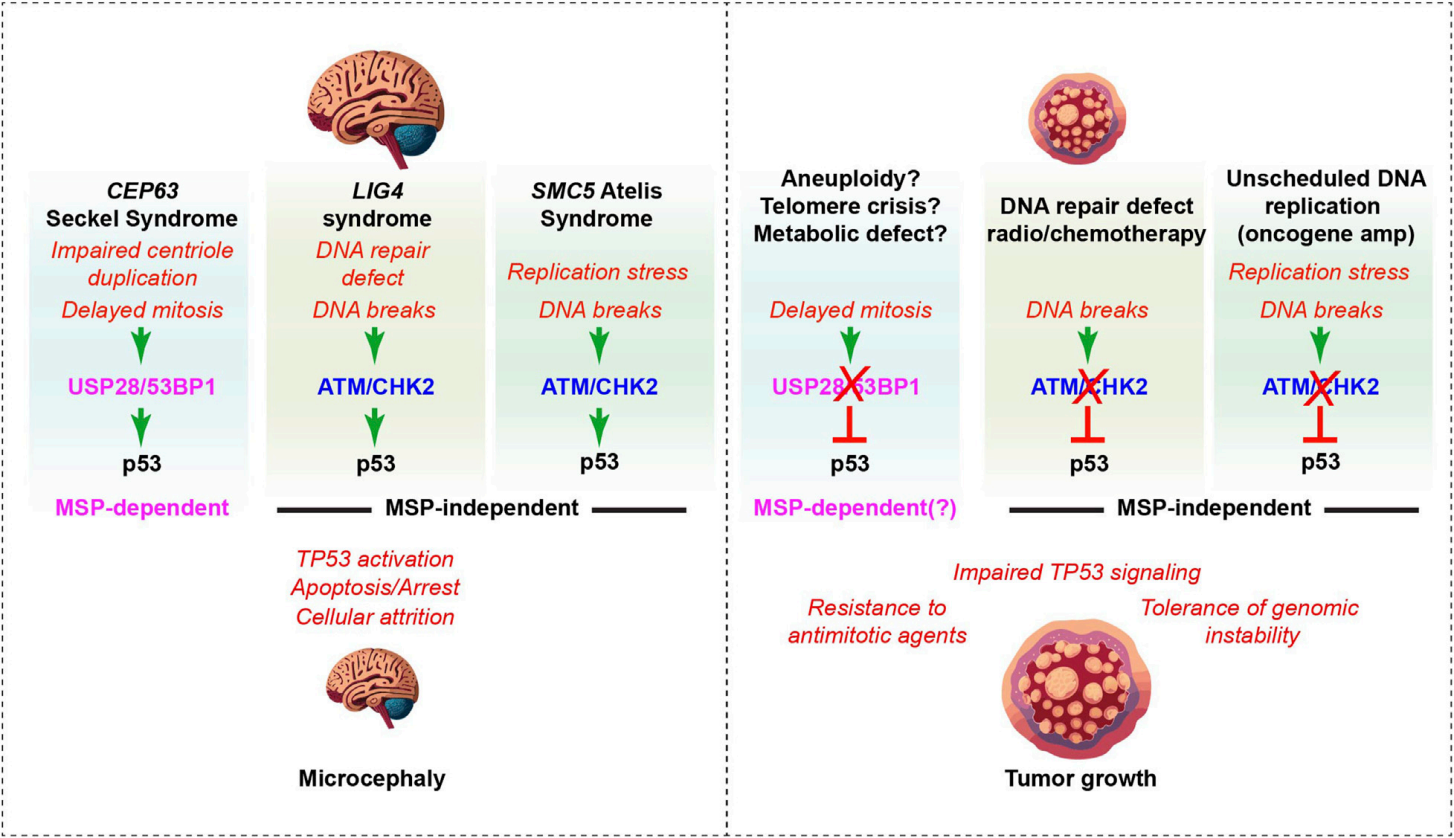
The MSP complex in neurodevelopment and cancer. Left panel: Delayed mitosis, DNA damage and DNA replication stress (red text), resulting from mutations in different genes involved in human neurodevelopmental disorders, activate p53 through distinct pathways, leading to cell death or arrest in mouse models (Marjanovic et al., 2015; Foster et al., 2012; Shull et al., 2009; O’Brien et al., 2023). The loss of p53 rescues NPCs and suppresses microcephaly phenotypes in many mouse models of human syndromes, including the 3 examples shown here (black bold text at the top of each column) (Bowen and Attardi, 2019; Marjanovic et al., 2015). MSP components USP28 and 53BP1 (pink) were selectively required for p53-activation and microcephaly in response to centrosome defects and delayed mitosis, as their absence failed to rescue DNA damage dependent microcephaly that was dependent on ATM/CHK2 DDR kinase signaling in other models (blue) (Atkins et al., 2020; Phan et al., 2021; Lee et al., 2000; Foster et al., 2012). Right panel: In cancer, frequent loss of USP28 is observed prior to treatment, including in some cancers with defective p53-transcriptional signatures, suggesting that there is a selective pressure for attenuating this component of the MSP (Fito-Lopez et al., 2023). Selection against MSP components could potentially result from aneuploidy, telomere crisis or metabolic defects that perturb mitotic progression, although it remains possible that MSP-independent functions of these proteins drive the selection for their loss (see text for discussion). MSP status predicts the response to antimitotic agents *in vitro* and likely should be considered in their clinical use (Meitinger et al., 2024). ATM and CHK2 loss predispose to cancer, likely due in large part to their role in activating p53 in response to DNA damage and replication stress. This allows tumors to tolerate higher levels of genomic instability that can drive the acquisition of aggressive traits and promote treatment resistance (middle and right column).

The activity of p53 has been implicated in several rare human syndromes characterized by microcephaly, or a smaller than normal head and brain at birth (Jayaraman et al., 2018). Microcephaly often arises due to the attrition of neural progenitor cells (NPCs) during development that can occur due to premature differentiation, cell death, cell cycle checkpoint arrest or senescence (Phan and Holland, 2021; Taylor et al., 2019). Many of the genes involved in these syndromes have defined roles in DNA replication/repair or mitotic cell division, the latter category being highly enriched for centrosomal proteins that are required for proper mitotic spindle formation (Jayaraman et al., 2018). While multiple lines of experimental evidence suggest that there are functional links between the DDR and centrosomes, recent work suggests that the MSP plays a key role in microcephaly arising specifically from centrosomal defects that slow mitotic progression (Figure 2) (Lambrus et al., 2016; Mullee and Morrison, 2016).

One of the first hints that centrosomal defects and prolonged mitosis could activate p53 through DDR-independent pathways *in vivo* came from the analysis of mice deficient for SAS4/CPAP that is mutated in Seckel syndrome (Phan and Holland, 2021). Conditional deletion of *Sas4* in the developing brain caused p53 activation and extensive cell death that could be rescued by p53 deletion (Bazzi and Anderson, 2014; Insolera et al., 2014). However, no spontaneous DNA damage was detectable and the DDR of *Sas4* knockout cells was intact (Bazzi and Anderson, 2014). Similar observations followed from the analysis of mice deficient for *Cep63,* also mutated in Seckel syndrome, that also developed microcephaly accompanied by elevated p53-dependent apoptosis (Marjanovic et al., 2015). Co-deletion of p53 rescued brain size, but deletion of either *Atm* or *Chk2* failed to modify the *Cep63* phenotype. As deletion of *p53*, *Atm* or *Chk2* rescued brain size in mice with DNA repair defects or replication stress, this indicated that the major stimuli was unlikely to be DNA damage, consistent with the lack of γH2AX or other DNA damage markers observed in the developing *Cep63* deficient brain (Figure 2) (Atkins et al., 2020; Marjanovic et al., 2015; Lee et al., 2000; Foster et al., 2012).

To determine if the MSP was the missing pathway responsible for triggering p53 activation in mice with centrosomal defects, a brain specific (*Nestin*-Cre) conditional knockout for *Sas4* and the *Cep63* deficient model were crossed with either *Usp28* or *53bp1* knockout mice (Lambrus et al., 2016). Homozygosity of either *Usp28* or *53bp1* loss reduced p53 activation and rescued brain size in both *Sas4* and *Cep63* microcephaly models (Figure 2). Notably, this occurred without rescuing centrosome duplication defects or miotic delays. In contrast to the *Sas4* and *Cep63* centrosome deficiency models, p53 activation and microcephaly caused by deletion of *Smc5*, that in mutated in Atelís syndrome and encodes part of the cohesin like SMC5/6 complex involved in DNA replication/repair, was rescued by loss of *Chk2* or *p53*, but not by *Usp28* or *53bp1* deficiency (Figure 2) (Atkins et al., 2020; Phan et al., 2021; Grange et al., 2022). These data established a role for the MSP in the neuropathology of a subset of microcephalic mouse models, and potentially human disorders, caused by centrosomal defects. Moreover, it identified distinct pathways that trigger p53-mediated cell death and microcephaly in response to either centrosomal defects and mitotic delays or DNA damage (Figure 2).

Early mouse embryos lack centrioles until embryonic day 3.5 (E3.5), indicating that mitotic spindle function and surveillance may differ during development (Courtois et al., 2012). In some centrosome mutants, for example, *Cep63* deficient mice, early embryonic lethality due to MSP activation has not been observed, suggesting that either its function is more essential for mitotic division in the brain or that the threshold to trigger the MSP is differentially established during early embryogenesis (Xiao et al., 2021). Knockout of *Sas4* was lethal by embryonic day 9.5 (E9.5) and embryos exhibited high levels of p53-induced apoptosis that was rescued by loss of either *Usp28* or *53bp1*, implicating MSP-mediated cell death in the embryonic lethality. A detailed analysis of embryogenesis revealed that the MSP was not active during development until E7, even though both *Usp28* and *53bp1* were expressed prior to E6. They examined centriole maturation and found that MSP activation correlated with centriole maturation, that occurred around E6.5. Whether centriole maturation itself influences the MSP remains unclear, but this work provided further evidence that the MSP plays a role in development and is inhibited at early stages, suggesting that additional regulatory inputs may exist and modify its function in different tissues. Whether TIRR, a well-established negative regulator of 53BP1, ERK signaling, that was identified as a key regulator of apoptosis in acentriolar lung cells, or other, yet to be identified regulatory mechanisms play a role in suppressing this response during development will be interesting to determine in future work (Xie et al., 2021; Botuyan et al., 2018).

Whether the MSP is responsible for microcephaly in human patients remains to be established. Studies using human brain organoids suggested that increased differentiation may be a more prominent mechanism of cellular attrition than apoptosis in humans (Gabriel et al., 2020). However, human brain organoids modeling a mutation in CENPJ/CPAP exhibited p53-dependent apoptosis, yielding smaller organoids (An et al., 2022). With future development of highly specific USP28 inhibitors and improved screening for microcephalic disorders, this could potentially be tested (Patzke et al., 2024). However, it is likely that rescuing brain size will not completely restore brain function. This aspect has not been meaningfully addressed in mouse models by behavioral analysis, but some abnormalities have been reported in size rescued brains (Insolera et al., 2014). Moreover, body size, that is affected to different extents in Seckel syndrome was not rescued by p53 loss in *Cep63*-deficient mouse models (Marjanovic et al., 2015). This suggests that additional, yet unidentified pathways respond to centrosomal defects in different tissues and impair growth in these mouse models, and potentially in human patients. One major candidate is primary ciliogenesis that may be differentially affected by distinct mutations that affect centrosome duplication and function (Phan et al., 2021; Gabriel et al., 2016; Wilsch-Brauninger and Huttner, 2021).

## The role of the MSP pathway in cancer

As the most well-established role of p53 is in tumor suppression, to what extent the MSP influences tumorigenesis remains an important question. 53BP1 is considered a tumor suppressor, as mice lacking 53BP1 were reported to develop lymphomas, albeit with a longer latency than p53 null mice (Ward et al., 2003). 53BP1 was identified as a haploinsufficient tumor suppressor in p53-deficient mice, suggesting p53-independent roles in tumor suppression (Ward et al., 2005). Haploinsufficiency of 53BP1 accelerated tumorigenesis in a mouse model of glioma that was similarly affected by the loss of ATM or CHK2, also implicating the DDR-dependent functions of 53BP1 in tumor suppression (Squatrito et al., 2012; Squatrito et al., 2010). Reduced expression of 53BP1 has been reported in different types of human cancer, including proneural Glioblastoma where around 35% exhibited the loss of a single copy of 53BP1 (Squatrito et al., 2012). Frequent 53BP1 loss of function mutations were also identified across cancer types, further supporting the proposition that it is a tumor suppressor (Mirza-Aghazadeh-Attari et al., 2019; Davoli et al., 2013).

USP28 was proposed to act as both an oncogene and a tumor suppressor (Prieto-Garcia et al., 2021). USP28 knockout mice were not predisposed to spontaneous tumorigenesis and conditional knockout of USP28 in the intestine did not alter intestinal morphology or lead to spontaneous tumors, although it reduced the numbers of some differentiated cell types in the crypt (Knobel et al., 2014; Diefenbacher et al., 2014). Loss of USP28 impaired tumorigenesis in the *Apc* ^*min*^ model of mouse colorectal cancer, concomitant with reduced levels of key oncogenic targets of FBW7, including c-MYC, NOTCH and JUN (Diefenbacher et al., 2015; Schulein-Volk et al., 2014). Despite the apparent necessity for USP28 function to drive some cancer types, multiple analyses of cancer genomes have proposed that USP28 is a tumor suppressor, like both p53 and 53BP1 (Davoli et al., 2013; Fito-Lopez et al., 2023). Analysis of tumors that phenocopied p53 loss at the transcriptional level, but had intact p53, identified amplifications of known p53 negative regulators, including *MDM2*, *MDM4* and *WIP1* (*PPM1D*), as an explanation in some cases (Fito-Lopez et al., 2023). In addition, deletions of *USP28* occurred in up to 7.6% of tumors that transcriptionally phenocopied p53 deficiency among several cancer types (Fito-Lopez et al., 2023). As USP28 plays an important role in activating p53 in response to mitotic delays, this could suggest that USP28 loss may be selected to tolerate some level of mitotic delay occurring in the genesis or evolution of these tumors (Figure 2). Consistent with this, cell lines with compromised or inactive MSP components showed impaired responses to anti-mitotic agents and the introduction of USP28 or 53BP1 loss of function alleles in the non-transformed RPE1 cell line led to defects in MSP function (Meitinger et al., 2024). Notably, the human tumors identified with USP28 mutations and p53-deficient transcriptional profiles were treatment naïve, ruling out potential selective pressure coming from the clinical use of antimitotic agents (Fito-Lopez et al., 2023).

Precisely what selective pressures drive the loss of 53BP1 and USP28 in cancer remains to be determined, but current data supports the proposition that MSP gene status could be used as a potential biomarker for poor responses to antimitotic drugs in cancer (Figure 2) (Meitinger et al., 2024). On the other hand, it could also suggest that the use of small molecule inhibitors to target USP28 in dependent cancers could have unintended effects through interference with the MSP and attenuation of some DNA damage induced p53 responses that should be considered.

## Discussion

Many questions remain as to how the MSP complex is regulated and how it activates p53-dependent transcription in response to distinct types of stress. Recent work established a role for direct phosphorylation of 53BP1 by PLK1 in the 53BP1 IDL domain. How these modifications alter the function of the IDL domain to promote MSP complex formation remains unknown. As mitotic phosphorylation of 53BP1 also prevents its activity in DNA repair until late mitosis, where it is reversed by PP4, it remains an open question whether this is integrated with the MSP (Orthwein et al., 2014; Zheng et al., 2019). Is extended time exposed to active PLK1 in mitosis sufficient to drive MSP phosphorylation events or is there a mediator of the signal between mitotic delays and PLK1 engagement of 53BP1? Is a specifically modified pool of p53 brought into these complexes or is a p53-methylase recruited to them? Is the catalytic activity of USP28 acting on p53 in assembled complexes to counteract its degradation? Do other post-translational modifications, such as SUMOylation of USP28 or methylation of 53BP1, or multimerization of USP28 or 53BP1, regulate the MSP (Jin et al., 2024; Zhen et al., 2014)? How is the transition from soluble MSP complex to DNA bound, transcriptionally active p53 initiated and how are MSP complexes maintained over cell cycles? Is phase separation, that regulates the role of 53BP1 in DNA repair involved (Zhang et al., 2022)? How distinct are the MSP complexes from p53-transcription complexes triggered by N3, DSBs or TIRR depletion? Future work will no doubt clarify many of these interesting questions and shed additional light on the molecular details of the control of the MSP pathway.

In the context of mammalian development, the MSP potentially plays an important role in a subset of microcephalic disorders caused by defects in centrosome function. In contrast to cells in culture that undergo p21-dependent arrest, p53 activation in the developing cortex results in apoptosis. While this difference in cell fate resulting from p53 activation is not unprecedented, it indicates that the cell fate outcome of MSP activation is dictated by cell type and microenvironmental context (Xie et al., 2021; Sullivan et al., 2018). Extending this to the organism, it will be important to determine how universally this pathway is used in order to understand its implications in other pathological outcomes of diseases where it is activated. For example, the deletion of p53 did not rescue the reduced body size of *Cep63* deficient mice, indicating that centrosome defects and mitotic delays may trigger additional pathways of cell arrest or cell death in other tissues during development (Marjanovic et al., 2015; Xiao et al., 2021).

The recent identification of USP28 loss in a significant number of p53-proficient tumors that exhibit p53-deficient transcriptional signatures suggests that the MSP may be selected against in a subset of cancers (Fito-Lopez et al., 2023). It is easy to imagine that mitotic defects caused by replication stress or telomere crisis, exogenous carcinogens that impair mitotic progression, or even metabolic defects that slow mitosis, could drive the selection of USP28/MSP loss (Figure 2) (Diehl et al., 2024). However, given that USP28 is also involved in transcriptional responses to DNA damage, as well as the stabilization of c-MYC and other oncogenes regulated by FBW7, it is difficult to determine precisely what selective pressure promotes its loss in these cancers without further information detailing the evolution of these cancers.

USP28 antagonizes FBW7 autocatalytic degradation in some tissues (Schulein-Volk et al., 2014). The outcome of this is that FBW7 targets are stabilized by both USP28 loss, due to the fact that FBW7 levels are reduced, as well as USP28 overexpression, due to FBW7 antagonism (Taranets et al., 2015). As many FBW7 targets play significant roles in cancer, this function of USP28 is likely relevant to its genetic alteration in some cancers and could certainly contribute to its roles as both a tumor suppressor and tumor promoter. FBW7 is highly mutated across cancers and leads to multidrug resistance (MDR), suggesting that this could be phenocopied by USP28 loss or amplification in some cancer types, although this this is not borne out in the CRISPR screening data that identified FBW7 in this role (Sanchez-Burgos et al., 2022). In addition, CRISPR screens implemented to identify genes that stabilize mutant p53 also identified USP28, although this role was independent of 53BP1 and dependent on CCDC6 and the FBXO42 ubiquitin ligase (Lu et al., 2024). USP28’s roles in regulating wild type and mutant p53, as well as FBW7, suggest again that some consideration of genomic context may be needed in the future implementation of USP28 inhibitors in cancer treatment. While there is clear data supporting this strategy in some cancer types, the impairment of p53-dependent cell fate decisions and FBW7 could be problematic, particularly if coupled with anti-mitotic agents.

Despite decades of study, new details of p53 regulation continue to emerge and there is no doubt that additional surprises will be uncovered in future work. Existing data clearly indicates that the MSP plays important roles in neurodevelopmental disorders and likely influences cancer etiology and cancer treatment responses. Additional work will be needed to untangle the regulatory mechanisms of the MSP and its relationship to additional pathways that use USP28 and 53BP1 to control cell function and fate through the regulation of p53.
